# Programmed Genome Elimination Is Evolutionarily Conserved Across *Pelophylax* Hybrids—As Evidenced by *P. grafi* Hybridogenetic Reproduction

**DOI:** 10.3390/biology14111526

**Published:** 2025-10-30

**Authors:** Anna Dudzik, Beata Rozenblut-Kościsty, Dmitrij Dedukh, Pierre-André Crochet, Lukáš Choleva, Monika Przewłocka-Kosmala, Zuzanna Stryczak, Maria Ogielska, Magdalena Chmielewska

**Affiliations:** 1 Amphibian Biology Group, Department of Evolutionary Biology and Conservation of Vertebrates, University of Wrocław, Sienkiewicza 21, 50-335 Wrocław, Poland; anna.dudzik@uwr.edu.pl (A.D.); beata.rozenblut-koscisty@uwr.edu.pl (B.R.-K.); maria.ogielska@uwr.edu.pl (M.O.); 2 Laboratory of Non-Mendelian Evolution, Institute of Animal Physiology and Genetics, Czech Academy of Sciences, Rumburská 89, 277-21 Liběchov, Czech Republic; dmitrijdedukh@gmail.com; 3 CEFE, CNRS, University Montpellier, EPHE, IRD, 1919, Route de Mende, 34293 Montpellier, France; pierre-andre.crochet@cefe.cnrs.fr; 4 Laboratory of Fish Genetics, Institute of Animal Physiology and Genetics, Czech Academy of Sciences, Rumburská 89, 277-21 Liběchov, Czech Republic; choleva@iapg.cas.cz; 5 Department of Biology and Ecology, Faculty of Science, University of Ostrava, Chittussiho 10, 710-00 Ostrava, Czech Republic; 6 Institute of Heart Diseases, Faculty of Medicine, Wroclaw Medical University, Borowska 213, 50-556 Wroclaw, Poland; monika.przewlocka-kosmala@umw.edu.pl

**Keywords:** hybridogenesis, *Pelophylax grafi*, amphibians, gametogenesis, genome elimination, micronuclei, gonocytes

## Abstract

**Simple Summary:**

Hybrids are organisms that result from the crossing of two different species. While plant hybrids are relatively common, like bread wheats and orchids, animal hybrids are rare and often sterile, because the chromosomes from the two parental species cannot pair correctly during the formation of eggs and sperm. Some animal hybrids, however, have developed unusual ways to reproduce. One such strategy is hybridogenesis, where the genetic material of one parent is selectively discarded in the cells that give rise to gametes. As a result, hybrids clonally transmit only the chromosomes from one parental species, while eliminating those of the other. The frog *Pelophylax grafi* is a natural hybrid between the Iberian water frog (*P. perezi*) and the marsh frog (*P. ridibundus*). To understand how this hybrid reproduces, we studied its germ cells at different stages of development. We found that the chromosomes of *P. perezi* are selectively eliminated before the formation of eggs and sperm, while those of *P. ridibundus* are retained and passed on to the next generation. This process explains how *P. grafi* maintains fertile populations in nature and reveals a conserved mechanism that may underlie reproduction in other hybrid animals.

**Abstract:**

Gametogenesis is a fundamental biological process that ensures both genetic recombination and the continuity of successive generations. Interspecific hybrids can reproduce through modified mechanisms, such as hybridogenesis, by transmitting clonal, unrecombined genomes of only one of the parental species via their gametes. *Pelophylax grafi* (RP) is a natural hybrid frog composed of mixed genomes (subgenomes) of two related species, *Pelophylax perezi* (P) and *Pelophylax ridibundus* (R), and coexists in populations with *P. perezi*. This study tested the involvement of programmed genome elimination in gamete production of *P. grafi*, providing new insight into reproductive mechanisms of hybrid vertebrates. Using comparative genomic hybridization (CGH) and fluorescent in situ hybridization (FISH), we examined the genomic constitution of germline cells in tadpoles and adult male and female *P. grafi*. Controlled crosses between *P. perezi* and *P. grafi* produced F1 hybrid tadpoles, whose genotypes confirmed that *P. grafi* parents transmitted the R subgenome through their gametes. In the early germline cells (gonocytes) of these tadpoles, P chromosomes were selectively eliminated via micronuclei formation during interphase. The occasional presence of the R genome and mixed R/P genome micronuclei suggests variability and imperfect fidelity in the elimination process. In adult hybrids, the majority of diplotene oocytes, spermatogonial stem cells (SSC) and spermatocytes carried R subgenomes. We demonstrated that programmed genome rearrangement in *Pelophylax* hybrids is an evolutionarily conserved mechanism underlying this unique reproductive strategy.

## 1. Introduction

Hybridogenesis is a reproductive strategy that consistently ensures the emergence of F1 hybrid individuals across successive generations. In the early germline cells (gonocytes) of these hybrids, one of the parental chromosome sets (subgenomes) is eliminated while the other is endoreplicated. This leads to the production of clonal (not recombined; asexual) gametes from one species and requires the contribution of recombined (sexual) gametes from the other species to renew the F1 hybrid progeny. Thus, hybridogenesis is a rare hemiclonal mode of reproduction, normally reliant on populations where both the hybrid taxa and at least one of its parental species coexist. It is observed in stick insects of the genus *Bacillus* [1], fish and amphibians [2,3,4]. Eight examples are documented among fishes, *Poeciliopsis lucida-monacha*, *Cobitis hankugensis* (*siniensis*)-*Iksookimia longicorpa*, *Misgurnus anguillicaudatus*, *Squalius alburnoides*, *Hexagrammos octogrammus*-*otakii* (-*agrammus*), *Hypseleotris* spp., and amphibians, *Bufotes pseudoraddei baturae*, *P. esculentus*, *P. hispanicus* and *P. grafi* [1,5,6,7,8,9,10,11,12,13,14,15]. However, the western Palearctic water frog group is a notable exception, containing two confirmed hybridogenetic hybrids, *P. esculentus* (*ridibundus-lessonae*, RL genome) and *P. grafi* (*ridibundus-perezi*, RP genome), which share the *P. ridibundus* (R) genome. Among them, *P. esculentus* is the most widespread taxon, possibly originally formed from crosses between (mostly male) *P. lessonae* (LL) and (mostly female) *P. ridibundus* (RR). *Pelophylax grafi* emerged in southwestern France and northeastern Spain, where the R genome was introduced not by the species *P. ridibundus*, but via the hybrid *P. esculentus*, which was expanding its range at the time [16,17,18,19]. Consequently, *P. grafi* is considered an evolutionarily younger hybrid. The contemporary literature on *P. grafi* does not address whether hybridization is currently occurring as a result of direct crossing between the parental species *P. perezi* and *P. ridibundus* [16]. In natural populations, *P. perezi* and *P. ridibundus* did not historically occur in sympatry; such a situation has only arisen in recent times [20].

Genome elimination occurs early in gametogenesis during gonad formation in developing tadpoles [21,22]. The earliest stage of gametogenesis in both sexes, known as pregametogenesis, is marked by the presence of specific precursor cells called gonocytes. In hybridogenesis, elimination of one subgenome and endoreplication of the other occur exclusively within these cells. Genome elimination occurs during gonocyte interphase [21,22,23,24]. The expelled chromosomes or their fragments are enclosed in micronuclei formed by budding off from the main nucleus. After detaching into the gonocyte cytoplasm, they undergo heterochromatinization and are degraded by nucleophagy, ensuring that only the non-eliminated parental genome remains to be duplicated and passed on through clonal gametes [21,24,25]. The gonocytes give rise to primary oocytes in females and spermatogonial stem cells (SSCs) in males [22]. Genome elimination has been extensively studied in *P. esculentus*, first described in juvenile hybrid females during oogenesis [26,27]. Cytogenetic studies on gametogenesis in adult *P. esculentus* females and males clearly showed which of the parental genomes was eliminated in early gonocytes and which genome was transmitted to the progeny [28,29,30,31,32,33,34,35]. *P. esculentus* males and females can follow multiple gametogenic pathways, producing a variety of gamete types (R, L, RL) within a single individual. As a result, mating between *P. esculentus* and *P. ridibundus* (R-E populations), *P. esculentus* and *P. lessonae* (L-E populations), or between two *P. esculentus* individuals (E-E populations) can give rise not only to hybrid offspring but also to progeny of the pure parental species—*P. ridibundus* and *P. lessonae*. This phenomenon has been observed in both laboratory crosses and natural amplexus pairs. However, the viability of such progeny is generally low [33,36,37,38,39]. In contrast, gamete production in *P. grafi* remains unstudied, and it is currently unknown whether *P. grafi* × *P. grafi* matings occur or whether the parental species—*P. perezi* or *P. ridibundus* can be restored in natural populations [16,17,18,19].

Cytogenetic studies on other hybridogenetic systems within the genus *Pelophylax*, namely *P. grafi* hybrid and its parental species *P. perezi*, were recently published [12]. Diploid somatic karyotypes of *P. perezi* and *P. grafi* have five pairs of large chromosomes and eight pairs of small chromosomes, similar to those of other *Pelophylax* taxa [12,40,41]. Karyotype analyses using genomic in situ hybridization (GISH) and comparative genomic hybridization (CGH) revealed that the *P. grafi* retains both parental chromosomal sets (subgenomes), confirming the F1 hybrid genome constitution [12]. The absence of visible homologous recombination blocks between the parental subgenomes in *P. grafi* confirmed hybridogenesis in this taxon, consistent with findings from previous studies on *P. esculentus* [40]. Similar results were obtained in a clonal fish, the Australian carp gudgeons *Hypseleotris* spp. [11,42]. FISH with *P. ridibundus*-specific pericentromeric RrS1 repeat showed similar distribution of this repeat among *P. ridibundus* and *P. perezi* chromosomes, which prevents their differentiation [12,34]. Nevertheless, *P. ridibundus* chromosome no. 10 has larger interstitial telomeric repeat sites flanking the nucleolus organizing region than the corresponding chromosome of *P. perezi*, enabling their recognition [12]. The analysis of lampbrush chromosomes from *P. perezi* revealed 13 fully paired bivalents [12], similar to those of *P. ridibundus* and *P. lessonae* [29,43]. A comparison of lampbrush chromosome morphology showed four distinct homology groups among *P. perezi*, *P. ridibundus*, and *P. lessonae*. Thus, the unique marker structure pattern observed in lampbrush chromosomes of *P. perezi* provides a useful tool for identifying the parental genome transmitted by *P. grafi*.

To examine possible mechanisms of genome elimination in *P. grafi*, we investigated gametogenesis across multiple ontogenetic stages. Based on the composition of *P. perezi*-*P. grafi* (P-G) populations and the karyotype of *P. grafi* [12], we hypothesized that the *P. perezi* (P) subgenome might be selectively eliminated in gonocytes, resulting in clonal transmission of *P. ridibundus* (R) chromosomes. This hypothesis had not been empirically tested, and we addressed it using CGH on chromosomal spreads from gonadal tissues of *P. grafi* tadpoles.

Our objectives were: (1) to confirm that the P subgenome is preferentially eliminated during pregametogenesis, (2) to confirm the micronuclei-mediated way of genome elimination from gonocytes, (3) to check whether the elimination and endoreplication are accurate by examining the genetic diversity of gonocytes and micronuclei in gonocytes of tadpoles, and (4) to confirm that the R subgenome is the one transmitted to functional gametes in adults. To address objectives 1 to 3, we examined qualitatively and quantitatively both the chromosomal composition of gonocytes and the type of genome eliminated by micronuclei in hybrid *P. grafi* tadpoles obtained by *P. grafi* × *P. perezi* and *P. perezi* × *P. ridibundus* in vitro crosses. We also wanted to determine whether hybridogenetic genome elimination and micronuclei formation occur in the offspring from matings between *P. perezi* and the invasive *P. ridibundus*, which cohabits with PG populations. To reach goal 4, we examined the genome composition of diplotene oocytes from hybrid females, as well as SSCs and spermatocytes from hybrid males. To determine whether adult hybrid males and females transmit only the R subgenome, we performed artificial crosses between two *P. grafi* individuals and genetically analyzed the genotypes of their offspring.

## 2. Materials and Methods

### 2.1. Animals and In Vitro Crosses

In this study, 23 adult frogs of both sexes were used. *P. perezi* and *P. grafi* were collected from three locations in southern France (Supplementary Table S1): Le Salagou River below the Dam du Salagou (43.6575, 3.4070; N = 14) and two locations near Montpellier, one from Creux de Miège pond (43.5230, 3.8195) and another from a pond near Rives d’Étang (43.506167, 3.811528), comprising one population (N = 6); the stream near El Ravaner, France (42.5198, 3.0550; N = 1). Among the 21 frogs from France 20 individuals were used for the in vitro crosses, including seven *P. grafi* females used additionally for lampbrush chromosome analysis, and one *P. perezi* female served for DNA extraction for the whole-genomic *perezi* probe. All individuals were collected in accordance with French legal regulations concerning wild species protection under permit number 2021-s-26 issued by the Direction régionale de l’environnement, de l’aménagement et du logement d’Occitanie. Two *P. ridibundus* males used for DNA extractions for preparation of the whole-genomic *ridibundus* probes were collected in Ruda Milicka in southwestern Poland (51.533153° N, 17.335117° E), in 2014 and 2016, following the permission of the Regional Directorate for Environmental Protection in Wrocław no. WPN.6401.82.2014.IW and no. WPN.6401.177.2016.IL.

Twelve families were raised in artificial crossing experiments conducted following established protocols for water frogs, as described by Berger et al. (1994) [44] and Chmielewska and Kaźmierczak et al. (2022) [45]. Twenty-four hours before the procedure, gravid females were injected intraperitoneally with salmon gonadotropin-releasing hormone (GnRH; also known as luteinizing hormone–releasing hormone, LHRH, H-7525.0001, Bachem, Bubendorf, Switzerland). After brief anesthesia in a 0.5% solution of ethyl 3-aminobenzoate methanesulfonate (MS-222, Merck Life Science, Poznań, Poland) in stale water H_2_O, the males were sacrificed by severing the spinal cord. Their body cavities were opened with an incision to remove the testes. One of the testes was subsequently homogenized for the insemination procedure, and the other was intended for cytogenetic studies. Tadpoles resulting from the in vitro crosses were reared in plastic tanks within a greenhouse, 7 individuals per 1 L, following the methods outlined by Berger et al. (1994) [44].

Eighty-two tadpoles resulting from six in vitro crosses, genotyped as *P. grafi* (Table 1, Supplementary Table S2), were intended for cytogenetic analysis of gonocytes from gonadal chromosomal preparations. Another set of 89 tadpoles deriving from seven different *P. grafi* × *P. grafi* backcrosses (Table 1, Supplementary Table S2) were genotyped by RFLP-PCR to identify genotypes of gametes passed on by the hybrid parents. Additionally, one wild-caught *P. grafi* tadpole was used for DAPI staining of the whole-mount gonads.

All the experiments mentioned above were conducted in compliance with national and international guidelines under the permits issued by the Local Commission for Ethics in Experiments on Animals in Wrocław, Poland (numbers 27/2016 and 040/2021/P1, 013/2024/P1) as an amphibian breeder listed by the Polish Ministry of Education and Science under number 054.

### 2.2. Taxonomic Identification

Taxonomic identification of adult frogs and tadpoles from experimental crosses was performed using RFLP-PCR, targeting species-specific SNPs within the recombination-activation gene 1 (*Rag1*) and tyrosinase 1 (*Tyr1*). Nuclear DNA was extracted from hindlimb soft tissue (adults, tadpoles at Gosner stages 40–45) or tails (tadpoles at stages 26–39) using the GeneMATRIX Tissue DNA Purification Kit (EURx, Gdańsk, Poland). PCR amplification followed protocols based on Cuevas et al. (2022) [46] and Dudzik et al. (2023) [12] with annealing temperatures of 58 °C for *Rag1* (C1000 Thermal Cycler, Bio-Rad, Warszawa, Poland) and 54 °C for *Tyr1* (Thermal Cycler, Applied Biosystems, Thermo Fisher Scientific, Waltham, MA USA). Enzyme digestion and fragment identification were performed following Cuevas et al. (2022) [46].

### 2.3. Preparation of Mitotic and Diplotene Chromosomes

Shortly before euthanasia, adult animals were anesthetized by immersion in a 0.5% aqueous solution of MS-222 (Merck Life Science, Poznań, Poland), and tadpoles were treated with a 0.25% solution. All adult hybrid males and all tadpoles from in vitro crosses destined for cytogenetic research were first sacrificed, their gonads dissected and incubated for 2–4 h at room temperature in 24-well dish with 1 ml of 0.0051% colchicine (Sigma Aldrich) in a culture medium containing: 54% L15 Leibovitz medium with glutamine (Thermo Fisher Scientific, Waltham, MA, USA); 36% deionized H_2_O; 10% fetal bovine serum (CytoGen, Zgierz, Poland); 1 U/mL penicillin, 1 mg/mL streptomycin (BioWest, Bradenton, FL, USA). After colchicine treatment, gonads were hypotonized in 0.075 M KCl for 10 min and fixed in cold Carnoy fixative (ethanol/glacial acetic acid in 3:1 proportion). Samples were stored at −20 °C until use.

To obtain chromosomal spreads, in the case of adult males, a small piece of gonadal tissue was placed in 70% acetic acid in the glass preparation chamber and homogenized using forceps under a stereomicroscope. For tadpoles, depending on the size of their gonads, between one and four pairs of gonads from the same cross and the same Gosner stage or with a difference up to 2 Gosner stages, were placed in the glass chamber and homogenized using forceps (Supplementary Table S3). The cell suspension was then spread over the microscopic slides placed on a sloped heating table at +60 °C. Afterward, the chromosomes on the slides were stained with the Giemsa solution for 5 min. The Giemsa-stained metaphase plates on slides were scanned by the Zeiss Axioplan epifluorescence microscope equipped with a CCD camera and ZEISS Axio Imager.2 epifluorescence microscope (Zeiss, Oberkochen, Germany) with Metafer platform (MetaSystems, Altlussheim, Germany). Selected slides were de-stained in ethanol series (50%, 70%, 96%) and used for CGH.

Lampbrush chromosomes were isolated from diplotene oocytes of seven *P. grafi* and one *P. perezi* females following a previously published protocol [47], with detailed descriptions for water frogs provided elsewhere [12,43]. Identification of marker structures and lampbrush chromosome maps was carried out according to Callan’s monograph [48] and previous studies on *P. perezi* and *P. ridibundus* [12,43].

### 2.4. Comparative Genomic Hybridization (CGH)

CGH was conducted following the protocol described in Dudzik et al. (2023) [12]. Genomic DNA from *P. perezi* and *P. ridibundus* was extracted from skeletal muscle and labeled via nick translation with biotin–dUTP and digoxigenin–dUTP (Abbott, Gretna, LA, USA), respectively (0.2 µg per slide). Fluorescent detection was performed using streptavidin–Alexa 488 (Invitrogen, Thermo Fisher Scientific, Waltham, MA, USA) for biotin and anti-digoxigenin–rhodamine (Invitrogen, Thermo Fisher Scientific, Waltham, MA, USA) for digoxigenin.

### 2.5. FISH with Telomeric Probe

For identification of the parental species genome transmitted in diplotene oocytes of *P. grafi*, we performed FISH with a telomeric probe following Dudzik et al. (2023) [12] on the lampbrush chromosome preparations.

### 2.6. Whole-Mount DAPI Staining

Gonadal tissues were dissected and fixed in 4% PFA in 1 × PBS at room temperature for 20 min. Following fixation, the tissues were washed three times with 1 × PBS for 5 min each to remove residual PFA. For permeabilization, tissues were incubated in 1 × PBS containing 0.1% Triton X-100 for 30 min at room temperature. After permeabilization, tissues were mounted on glass slides with Vectashield medium containing DAPI (1.5 mg/mL) (Vector, Newark, CA, USA) and coverslipped. Samples were imaged using a Leica TCS SP5 confocal microscope based on the inverted microscope Leica DMI 6000 CS (Leica Microsystems, Wetzlar, Germany). A diode laser was used to excite the DAPI. Images were captured and processed using the associated LAS AF software (version: 4.0.11706, Leica Microsystems, Germany).

### 2.7. Analysis of Mitotic Chromosomes, Interphase Gonocytes, Micronuclei in Gonocytes and Pre-Diplotene Meiocytes

When tadpoles in slightly different Gosner stages were used for the same slide, the average Gosner stage (rounded to the nearest whole number) was recorded for the group. Subsequently, all tadpoles were divided into grouped Gosner stages into six categories: 28–30; 31–33; 34–36; 37–39; 40–42; 43–45, to ensure data unification and avoid small sample sizes. The classification process for metaphase plates was crucial for assessing the quality and quantity of chromosomes and analyzing their fate. Each metaphase plate was examined, and the total chromosome number, as well as the presence of large and small *perezi* (P) and *ridibundus* (R) chromosomes, were recorded. Data from each slide were documented in Excel spreadsheets (Supplementary Table S4). Since the chromosome numbers varied between plates and did not always correspond to the euploid number, we assumed that a haploid set (1n) ranged from 11 to 13, a diploid set (2n) from 24 to 26, and 36–39 as a triploid (3n). Hypohaploid metaphase plates with fewer than or equal to 10 chromosomes were excluded from statistical analysis.

The pre-diplotene meiocytes were analyzed qualitatively, focusing on the type of subgenome present (Supplementary Table S6). The micronuclei were categorized into three groups: (1) those containing only the P subgenome; (2) those containing only the R subgenome; and (3) those containing both mixed R/P subgenomes (Supplementary Table S5).

### 2.8. Statistical Analysis

The data from chromosome counting in metaphase plates were imported into Google Colaboratory and processed in Python 3.10 using the pandas, matplotlib, and seaborn libraries, with code-writing support from ChatGPT-4o and ChatGPT-5 (OpenAI). Data manipulation steps included aggregation of counts by cross and by parental genotype. For exploratory analysis, we applied multiple visualization approaches: bar charts and lollipop charts to display distribution and frequencies of chromosome numbers and micronuclei count, violin plots to show variation and spread, and heatmaps to compare patterns of germline cell ploidy and micronuclei within crosses and in four hybrid adult males. These plots allowed preliminary evaluation of each cross, after which we grouped the crosses into three categories according to parental genotype for further comparison.

Further analysis was performed using standard statistical software (Statistica version 13.3, TIBCO Software Inc., Palo Alto, CA, USA). We analyzed individual values of R and P chromosome content for each metaphase from six crosses used for CGH, restricted to 34–45 Gosner stages (Supplementary Tables S8–S10). To quantify the elimination of P chromosomes across developmental stages, we calculated the rate of decline (drop rate, DR) for each individual. DR represents the percentage decrease in the average number of R or P chromosomes relative to the initial haploid number (1n = 13), according to the formula: DR = 100% × (13 minus the average number of chromosomes at a given Gosner stage). Data expressing DR are presented as mean ± SD in each family (6 subsets/crosses) and after grouping by maternal R chromosome inheritance (2 subsets). Since the analyzed variables were not normally distributed, non-parametric tests were used for intergroup comparisons: the Mann–Whitney U-test in the 2-subset analysis and the Kruskal–Wallis test in the 6-subset analysis. The Wilcoxon matched-pairs test was used to compare the drop rates of large and small P chromosomes within subgroups based on the paternal or maternal origin of R chromosomes. Associations between the rate of decline in the number of chromosomes and the presence of maternal R chromosomes were assessed by univariable regression analysis. *p* values < 0.05 were considered significant.

### 2.9. Image Processing

Chromosome preparations were analyzed by a Zeiss Axioplan epifluorescence microscope equipped with a CCD camera and ZEISS Axio Imager.2 epifluorescence microscope (Zeiss, Oberkochen, Germany). Slides were scanned using a 10× objective with Metafer scanning software (MetaSystems, Altlussheim, Germany). Images of metaphase plates were recorded with a CoolCube 1 camera (MetaSystems, Altlussheim, Germany). To analyze gray-scale images, IKAROS and ISIS imaging software (MetaSystems, Altlussheim, Germany) were used. Chosen images were then adjusted with the Krita 5.1.5 software.

### 2.10. Use of Artificial Intelligence Tools

Some portions of the manuscript text—such as translation from Polish to English, language refinement, and shortening of descriptions—were assisted by ChatGPT-4o (OpenAI) based on author-provided input. All content was reviewed, edited, and approved by the authors.

## 3. Results

### 3.1. Chromosomal Composition of Gonocytes and Oocytes in P. grafi Tadpoles During Gonadal Development (Detailed Description in Supplementary File S1)

The hybrid progeny (N = 82 tadpoles) was obtained from six in vitro crosses (Table 1) divided into three groups according to the parental genotypes described in the paragraphs below. RFLP-PCR analysis revealed that all examined tadpoles exhibited the *P. grafi* genotype, as expected from parental identity (Supplementary Table S2).

**Figure 1:**
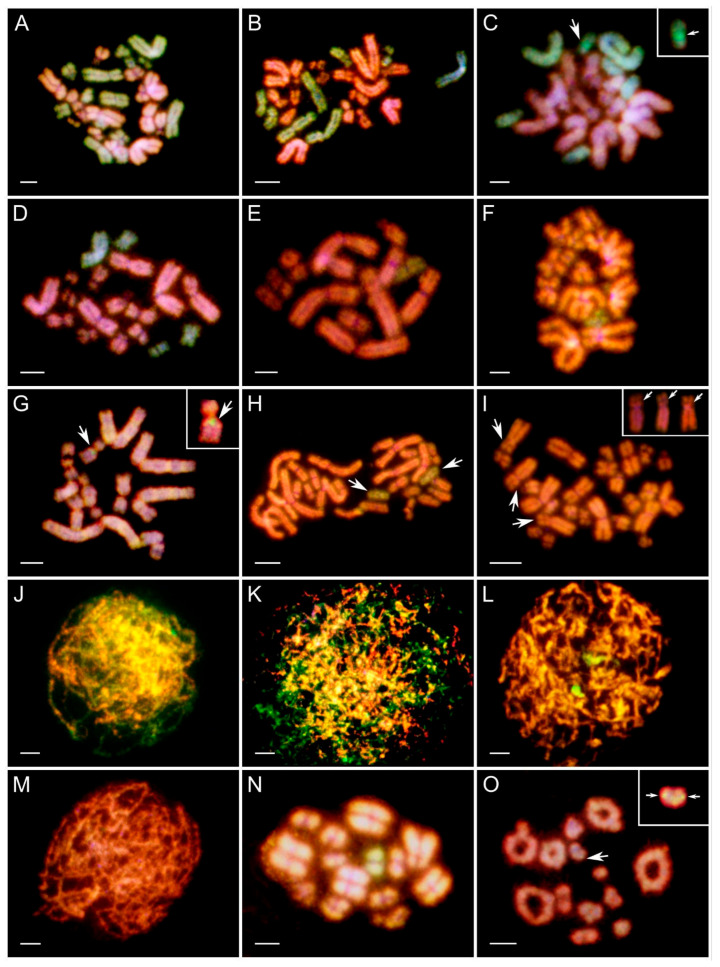
Comparative genomic hybridization on mitotic (**A**–**I**) and meiotic (**J**–**O**) gonadal spreads of tadpoles obtained from in vitro crosses. (**A**–**D**,**F**–**O**)—female tadpole ovaries, (**E**)—tadpole male testes. *P. ridibundus* (R) chromosomes—red, *P. perezi* (P) chromosomes—green. Chromosomal compositions of gonocytes: (**A**) 13 R and 13 P chromosomes; (**B**) 13 R and 8 P chromosomes (4 big, 4 small); (**C**) 13 R and 7 P chromosomes (2 big and 5 small), among which small 12th P chromosome (pointed by large arrow) has bright-green pericentromeric region (indicated by small white arrow in inset); (**D**) 13 R and 4 P chromosomes (1 large, 3 small); (**E**) 12 R and 1 small P chromosome (no 6 or 7), male tadpole’s gonad; (**F**) 13 R and 1 P chromosomes (from pair 6, 7 or 11); (**G**) 13 R chromosomes, among which small R chromosome (large arrow) bears green pericentromeric signal of *perezi* probe (small white arrow in the inset); (**H**) a diploid set of 26 R chromosomes with an additional two small P chromosomes pointed by white arrows (pair 6 or 7); (**I**) diploid 26 R metaphase plate with three big chromosomes (first two from the 2nd pair, third from the 3rd pair) excluded in the inset with possible double chromosomal breakage on their p-arms (pointed by arrows). Meiotic figures obtained from female gonads: (**J**) leptotene/zygotene stage oocyte with mixed genomes; (**K**) leptotene/zygotene mixed genome oocyte with highly dispersed chromatin of meiotic prophase I chromosomes; (**L**) nearly purely R zygotene/pachytene oocyte with two green spots of P whole-genomic probe; (**M**) pure R leptotene/zygotene oocyte; (**N**) 26 R and 2 small P meiotic chromosomes forming bivalents; (**O**) 13 R diplotene bivalents, one small with two green spots located in the pericentromeric region (pointed by white arrows, in the inset). Figures (**A**,**B**,**D**,**J**)—cross 6 and 30, (**E**,**H**,**I**,**K**,**L**–**O**)—cross 18 and 24, (**C**,**G**)—cross 19, (**F**)—cross 12. Scale bars 10 µm.

*Chromosomal compositions of mitotic gonocytes in all crosses*. We analyzed 460 gonocytes’ metaphase plates (Supplementary Tables S2–S4). In the youngest tadpoles from the Gosner stage 28 (cross 18) and 31–33 (cross 6), diploid RP chromosomal sets with 13 chromosomes of *P. ridibundus* and 13 chromosomes of *P. perezi* were found in most metaphase plates (Figure 1A). Starting from stage 34, the number of R chromosomes remained constant (13 or 12), while the number of P chromosomes began to decrease, resulting in hypodiploid plates (Figure 1B–F). We interpreted them as cells during the selective P subgenome elimination. In crosses 18 and 24 at stages 34–36 and 37–39, almost all metaphase plates had 3 or fewer P chromosomes, including haploid R (Figure 1G), diploid R (Figure 1I) and hypotriploid R chromosomal sets (Figure 1H). At stages 31–33 and 37–38 from cross 12, the elimination of the P genome was very weakly pronounced, and hypodiploid metaphases were in the minority (Figure 1F, one P chromosome). In stages 39–44 from crosses 6 and 30, haploid plates with 13 R chromosomes and diploid plates with 26 R chromosomes were observed for the first time, indicating that these crosses exhibited slower elimination and endoreplication compared to crosses 18 and 24. In the most advanced tadpoles at Gosner stages 40–42 and 43–45 from crosses 18, 19, and 24, we found that one-fourth of the cells contained only the R genome, including both haploid and diploid chromosome sets. One-fifth of the metaphase plates were diploid RP, primarily originating from tadpoles in cross 19. This cross showed a slower rate of P chromosome elimination, like crosses 6 and 30. As a result, statistical analysis for cross 19 was performed separately from crosses 18 and 24. In crosses 18 and 24, except for most of the female tadpoles, we also identified three male tadpoles. Gonocytes from male testes also showed selective elimination of the P genome (Figure 1E). However, the data from these males were not included in the charts or statistical analysis. Across all stages in crosses 6, 18, 24, and 30, we observed rare mitoses (*n* = 25) with fewer than 12 R chromosomes, suggesting non-selective elimination of the R genome. Additionally, some metaphase plates showed reduced numbers of both P and R chromosomes, indicating elimination of both subgenomes.

*Characteristics of metaphase chromosomes*. Among all crosses analyzed, we found no evidence of large intergenomic introgressions in most chromosomes of the R and P genomes. However, introgression of the P genome was frequently observed in the pericentromeric region of the short arm on one of the small R chromosomes, most likely from pair 12 (as presented in the inset in Figure 1G for cross 19). Such an enhanced signal was also visible on the small 12th P chromosome (inset in Figure 1C). Introgression cases were identified in crosses 6 and 30 (*n* = 22), in crosses 18, 19 and 24 (*n* = 44), and in cross 12 (*n* = 2). Additionally, we observed large chromosomes with possible chromosomal breaks (Figure 1I) in metaphase plates (*n* = 38) from all crosses except cross 12.

#### 3.1.1. Female *P. perezi* PP × Male *P. grafi* RP

Crosses 6 and 30 involved *P. perezi* females (nos. 532 and 537) and *P. grafi* males (nos. 546 and 545), all originating from the Le Salagou population. Tadpoles were analyzed at stages 31–45 (N = 44; 216 metaphase plates, Supplementary Tables S2–S4). Most tadpoles were females (*n* = 42) except two tadpoles with sexually undifferentiated gonads at stage 31.

**Figure 2:**
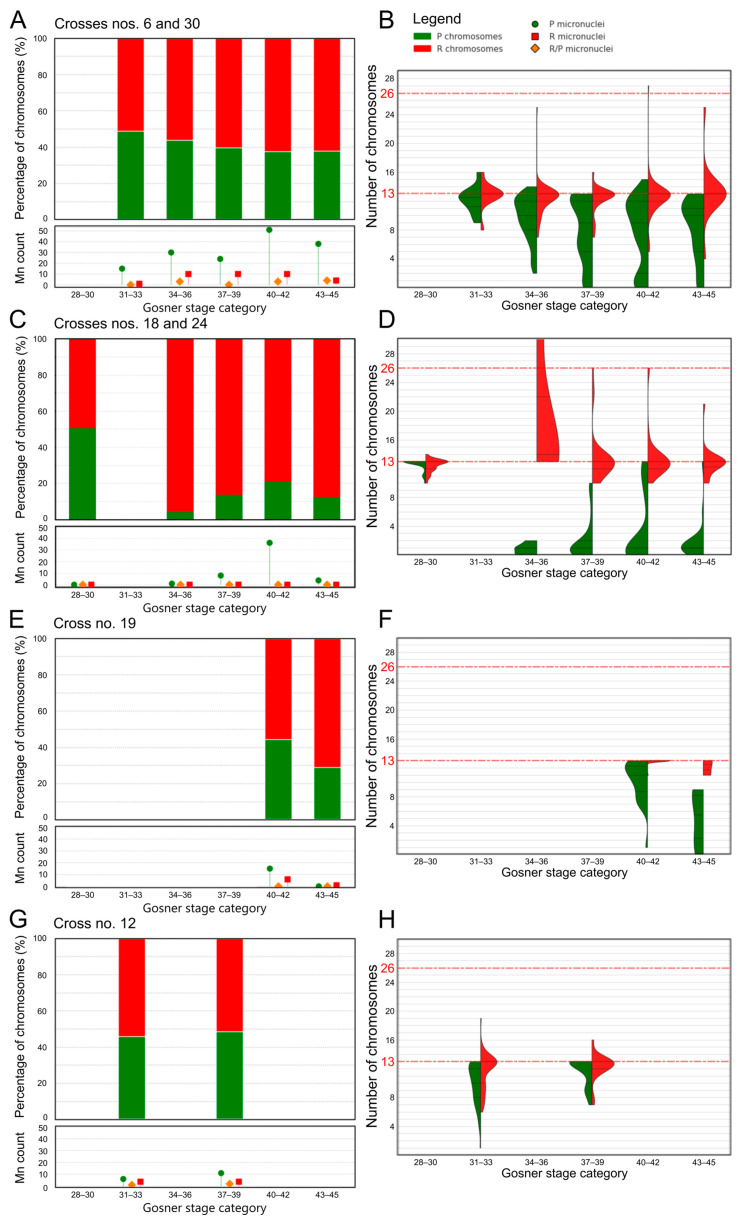
Chromosomal distribution in gonadal spreads of tadpoles from in vitro crosses. (**A**,**C**,**E**,**G**)—percentage distribution of *perezi* (P, green) and *ridibundus* (R, red) chromosomes across different Gosner stages (28–45). Below each bar chart, lollipop plots show the raw counts of micronuclei with P, R, and mixed (P/R) genotypes. (**B**,**D**,**F**,**H**)—violin plot comparisons of P and R chromosome count across Gosner stages. Dashed red lines indicate haploid (n = 13) and diploid (n = 26) chromosome numbers. Panel B contains the legend for all plots. (**A**) Crosses 6 and 30: the proportion of P chromosomes gradually decreases with advancing Gosner stages, while micronuclei count increase in the middle stages and decline in the final category. (**B**) Violin plots for crosses 6 and 30: R chromosomes remain stable around 13 with low variability, while P chromosomes decrease and show broader distributions, indicating higher variability across mitoses. (**C**) Crosses 18 and 24: after stage 28, the percentage of P chromosomes drops sharply, then slightly rises in later stages without a clear trend. (**D**) Violin plots for crosses 18 and 24: R chromosomes remain stable near 13 except at stages 34–36, where a single value reaches 35, exceeding the plot’s window. P chromosome counts are markedly lower, with greater variability at stages 40–42. (**E**) Cross 19: only two stage categories were distinguished; in both, P chromosome percentages are lower than R. (**F**) Violin plots for cross 19: R chromosomes remain near 13; P chromosomes are consistently lower, with broader distributions in both categories. (**G**) Cross 12: no clear trend in P chromosome percentages is observed across stages. (**H**) Violin plots for cross 12: R chromosome counts are stable at 13, with slightly greater variability at stages 31–33. P chromosome counts are lower, with visibly reduced medians compared to R chromosomes.

*Frequency of R and P chromosomes in gonocytes and the rate of genome elimination*. In pooled mitotic gonocytes at stages 31–33, R chromosomes constituted an average of 51.20%, and P chromosomes reached 48.80% (Figure 2A). The percentage of P chromosomes gradually decreased as development progressed, reaching the minimum of 37.95% at stages 43–45. The violin plot for the P chromosome section confirmed a declining trend, while the R chromosome section showed a relatively constant count of 13 chromosomes, although a few counts fell below this number (Figure 2B). As development progressed, the spread of the data increased in the violin plot for P chromosomes, indicating increased variability and suggesting selective loss of P chromosomes. Three diploid RR metaphase plates witnessed successful endoreplication of R chromosomes. Based on these data, we determined that crosses 6 and 30 exhibited a moderate rate of P genome elimination.

**Figure 3:**
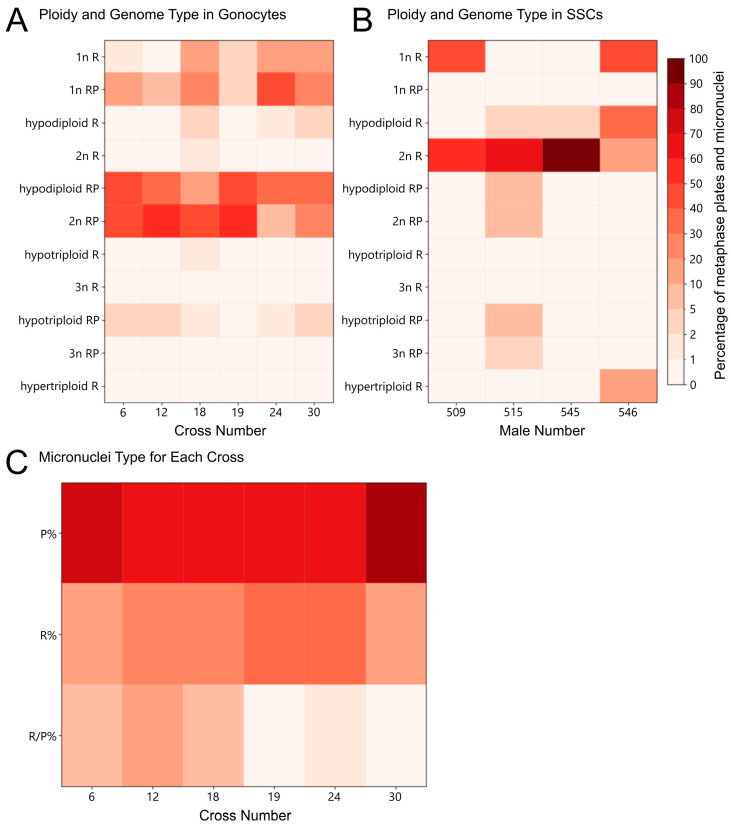
Ploidy types and genomic compositions of gonocytes, their micronuclei in tadpoles, and SSCs in adult males. (**A**) Heatmap of ploidy levels and genomic compositions of metaphase plates across different hybrid crosses. (**B**) Heatmap of ploidy types and genomic compositions of SSCs in individual males. (**C**) Heatmap showing the percentage composition of micronuclei types (P—*perezi* genome, R—*ridibundus* genome, R/P—genomes from both species), grouped by in vitro crosses and Gosner stage categories. The color scale (see legend) represents the percentage of metaphase plates and micronuclei. The Y-axis represents the percentages, while the X-axis indicates the number of in vitro crosses or adult males. Colors correspond to ploidy and micronuclei percentages, as indicated in the legend.

Among ploidy levels and genomic compositions of gonocytes, the most common were diploid RP (42.76–22.45%) and hypodiploid RP metaphase plates (41.38–36.73%), corresponding to the cells before and during genome elimination (Figure 3A, Supplementary Tables S5 and S6). Cells with a haploid R genome after successful elimination of the P genome (1.38–12.24%), or with a hypodiploid or hypertriploid RR genome after endoreplication (0.69–2.04%) were less frequent. The next subset of cells was aneuploid with the R genome or mixed RP genomes varying from haploid to triploid chromosome sets.

*Pre-diplotene meiotic prophase I oocytes*. Among 24 oocytes (up to pachytene) (Supplementary Table S7), we recorded mixed R/P genomes (*n* = 19, Figure 1J), mainly R chromosomes with a few P chromosomes (*n* = 1, Figure 1L), and exclusively R chromosomes at Gosner stages 39 and 43 (*n* = 4, Figure 1M). Three of the mixed R/P oocyte nuclei displayed highly dispersed chromatin threads (Figure 1K).

#### 3.1.2. Female *P. grafi* RP × Male *P. perezi* PP

In crosses 18 and 24, each parental pair originated from the same population: *P. grafi* females (nos. 503 and 531) and *P. perezi* males (nos. 510 and 548) were from Montpellier in cross 18, and from Le Salagou in cross 24. Among 23 tadpoles, 16 were females, three were males, and four individuals were sexually undifferentiated. Cross 19 involved parents from different populations: the *P. grafi* female (no. 520) from Le Salagou and the *P. perezi* male (no. 510) from Montpellier. The progeny of this pair included six female tadpoles analyzed at stages 28–45. Altogether, we analyzed 29 tadpoles from the above crosses (167 metaphase plates, Supplementary Tables S2–S4).

*Frequency of R and P chromosomes in gonocytes*. The tadpoles from crosses 18 and 24 rapidly eliminated P chromosomes. The proportion of P chromosomes reduced rapidly from nearly equal to R chromosomes (50.32% R and 49.68% P) at stage 28 to 3.96–21.11% in the following stages (Figure 2C,D). In contrast, the number of R chromosomes was relatively constant (13 R), although a few counts fell 1–3 chromosomes below this value. Notably, the stage category 34–36 exhibited a significantly higher number of R chromosomes, accompanied by a widened range of values in the violin plot, indicating greater variability in counts and ongoing endoreplication of the R subgenome. For the P chromosome section, the spread of the data increased in the violin plot in category 40–42. In the remaining stage categories, the range was narrow, with stage 28 showing values oscillating around 13 chromosomes, while in other stages, it ranged from 0 to 2, with only a few counts exceeding this range (Figure 2D). The data above suggest that crosses 18 and 24 displayed a fast rate of P genome elimination, compared to crosses 6 and 30.

The tadpoles from cross 19 had a more stagnant genome elimination process compared to crosses 18 and 24. Stage category 40–42 had quite a high P chromosomes percentage, equal to 44.31%, which decreased to 28.98% as the development progressed (Figure 2E). The violin plot for cross 19 presents the gradually decreasing P chromosomes number as the stages follow, with high count variability. In the tadpoles from this cross, we did not observe diploid RR chromosomal complements after endoreplication (Figure 2F).

We identified various ploidy levels and genomic compositions of gonocytes in crosses 18, 19 and 24. Diploid RP metaphase plates mostly prevailed (5.08–51.61%), followed by hypodiploid RP compositions (10.94–45.16%) (Figure 3A, Supplementary Tables S5 and S6). Metaphases with haploid R genomes (16.95–17.19%) were more frequent than in crosses 6 and 30, while diploid cells with RR genome (1.56%) were rare. Additionally, the aneuploid cells with mixed RP or R genomes of various ploidy levels constituted a substantial fraction.

*Pre-diplotene meiotic prophase I oocytes*. Oocytes in meiotic prophase I first appeared at stage 38 (Supplementary Table S7). We recorded 92 oocytes: 25 contained mixed R/P genomes (Figure 1J), 31 carried mainly the R genome with a few P chromosomes (Figure 1L), and 36 from cross 24 at stages 38–45 contained only R chromosomes (Figure 1M; Supplementary Table S6). Three meiotic figures displayed pachytene bivalents: two had 13 R and 1 P bivalents, and one had 13 R bivalents (Figure 1N,O). Five mixed R/P meiocytes displayed highly dispersed chromatin threads (Figure 1K).

#### 3.1.3. Female *P. perezi* PP × Male *P. ridibundus* RR

Cross no. 12 was a primary interspecific cross between parental individuals originating from distinct populations: a *P. perezi* female (no. 521) from Le Salagou and a *P. ridibundus* male (no. 513) from the Montpellier population. The tadpoles obtained from this cross, all female, were analyzed at stages 31–38 (N = 9; 77 metaphase plates, Supplementary Tables S2–S4).

*Frequency of R and P chromosomes in gonocytes*. We found similar percentages of P and R chromosomes in stages 31–33 (45.74% of P and 54.26% of R) and 37–39 (48.40% of P and 51.60% of R) stage categories (Figure 2G). No apparent trend of the P genome elimination was observed. The number of P chromosomes across stage categories (Figure 2H) was generally stable, without a clear trend toward reduction. The number of R chromosomes (Figure 2H) also remained constant, close to the haploid composition. The violin plot remains relatively short across both stage categories, implying limited variability of R chromosome numbers throughout development (Figure 2H).

Analysis of the gonocytes ploidy in cross 12 revealed 50.67% diploid RP, 9.33% haploid RP, 36.00% hypodiploid RP, and 4.00% hypotriploid RP metaphase plates (Figure 3A, Supplementary Tables S5 and S6).

*Pre-diplotene meiotic prophase I oocytes*. A total of 10 pre-diplotene meiotic oocytes were recorded, all of them having mixed R/P genotype (Supplementary Table S7).

#### 3.1.4. Statistical Summary of All the Crosses Pooled

We performed the statistical analysis on 271 metaphase plates from female tadpoles at stages 34–45 described above, excluding sets below 11 chromosomes, to provide a more accurate representation of chromosomal drop rate dynamics. The Kruskal–Wallis ANOVA revealed a statistically significant difference in the drop rate percentage of P chromosomes (P% drop rate) among the crosses (*p* < 0.001), but not for the R chromosomes (R% drop rate) (Supplementary Table S8). We also divided and counted the big and small P and R chromosomes. The descriptive statistics for big (big P% drop rate) (*p* < 0.001) and small (small P% drop rate) (*p* < 0.001) P chromosomes showed differences within the groups. Analysis using the Kruskal–Wallis ANOVA identified specific intergroup differences for the P% drop rate among all six analyzed crosses (details in Supplementary File S1 and Supplementary Table S8). Crosses 18 and 24 showed a higher drop rate (mean drop rate P% range 1.897–2.126) in relation to other crosses (mean drop rate P% range 0.307–1.289), indicating they are more prone to P chromosome elimination.

Next, we conducted descriptive statistical analysis for two subgroups based on the origin of the R subgenome (Supplementary Table S9) in tadpoles, either inherited from the maternal side (nos. 18, 19 and 24) or from the paternal side (nos. 6, 12 and 30). The subgroup with a maternal *P. grafi* (RP) origin of the R genome exhibited a higher P% drop rate (mean of 1.44 ± 0.99) compared to the paternal *P. grafi* (RP) subgroup (mean of 0.99 ± 0.87). For the R% drop rate, the maternal *P. grafi* (RP) group had a mean of −0.01 ± 0.51, while the paternal *P. grafi* (RP) group had a mean of 0.09 ± 0.50. The Mann–Whitney U test demonstrated significant differences for the P% drop rate (*p* < 0.001), but not for the R% drop rate (*p* = 0.598), indicating that the parental origin of the R subgenome affects the rate of P genome elimination (details in Supplementary File S1). The Mann–Whitney U test for big P% chromosomes drop rate showed a significant difference (*p* = 0.002), suggesting that the maternal origin of R chromosomes results in faster elimination of the P subgenome in tadpole gonads. Wilcoxon matched pairs test revealed statistically significant differences in the rate of decline between large and small chromosomes in the group with maternal origin of R chromosomes: 1.48 ± 1.12 for big P% drop rate vs. 1.42 ± 0.95 for small P% drop rate (*p* = 0.006) (more details in Supplementary File S1). Additionally, univariable regression analyses were performed to assess the association between the P% chromosomes drop rate and the origin of the R genome in tadpoles (Supplementary Table S10). A significant positive association was detected (F (1,269) = 15.307, *p* < 0.001), with a multiple R of 0.23 and adjusted R^2^ of 0.05. This means that the drop rate of P chromosomes increases when R chromosomes are maternally inherited. For the R% drop rate, no significant association was observed (F (1,269) = 2.775, *p* = 0.097), with a multiple R of 0.10 and an adjusted R^2^ of 0.0065 (more details in Supplementary File S1).

### 3.2. The Characteristics of Micronuclei in Gonocytes

**Figure 4:**
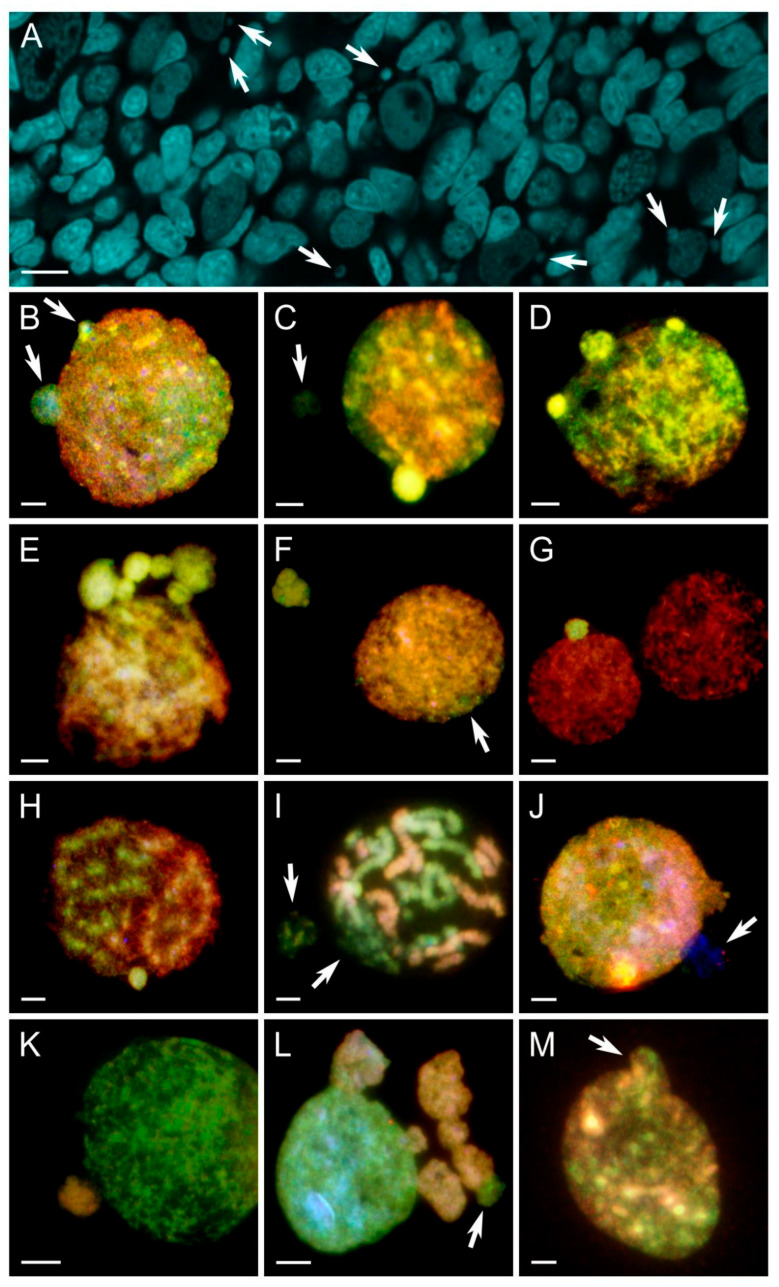
Micronuclei formation and their genome composition in hybrid gonocytes differentiated with comparative genomic hybridization. *P. ridibundus* (R) chromatin—red, *P. perezi* (P) chromatin—green. (**A**) whole-mount DAPI-stained gonadal tissue fragment of *P. grafi* tadpole ovary with multiple micronuclei (pointed by arrows). (**B**) mixed genome interphase nucleus with two budding micronuclei with the P subgenome. (**C**) mixed genome interphase nucleus showing one budding P micronucleus with bright green fluorescence indicating heterochromatinization and one P micronucleus with a faint signal. (**D**) mixed genome interphase nucleus with three budding P micronuclei, two of which exhibit bright green fluorescence, indicating heterochromatinization. (**E**) interphase nucleus predominantly with R subgenome (red) with 6 distinct P micronuclei. (**F**) nearly pure R interphase nucleus, with P signal on the edge (white arrow) and one irregularly shaped P micronucleus. (**G**) two pure R interphase nuclei with one P micronucleus present. (**H**) mitosis at prophase with segregated genomes of both species with one P micronucleus. (**I**) mitosis at prometaphase with decondensed P chromatin and P micronucleus. (**J**) mixed genome interphase nucleus with two micronuclei: one R and one only stained with DAPI. (**K**) P interphase nucleus with one R micronucleus. (**L**) P interphase nucleus with 6 R and one P micronuclei, all interconnected either to each other or to the main nucleus and with irregular shapes. (**M**) mixed R/P interphase gonocyte with a budding micronucleus with a mixed genome composition. Scale bars 10 µm.

*Types of genomes identified within micronuclei*. Whole-mount DAPI staining of the tadpole ovary revealed the presence of micronuclei enclosed in gonocyte cytoplasm (Figure 4A). Micronuclei were also found in interphase cells on the chromosomal preparations (Figure 4B–M). We examined 295 interphase gonocyte nuclei with their accompanying 332 micronuclei (Supplementary Table S11). Micronuclei emerged by budding from the main nucleus (Figure 4B–D,G,J,L,M). In two cases, the micronuclei were present near the nuclei entering mitosis (Figure 4H,I); in one prometaphase, a portion of P chromosomes was decondensed (Figure 4I). The interphase nuclei mainly contained mixed R/P subgenomes with varying degrees of chromatin compartmentalization (Figure 4B,C,J,M). We also observed gonocytes containing predominantly the R genome (Figure 4E,F), as well as only the R genome (Figure 4G), witnessing ongoing or completed elimination of the P subgenome. Two gonocytes nuclei contained only the P genome (Figure 4K,L), while their micronuclei displayed R chromatin signal. In a gonocyte from cross 19, we found six R micronuclei and one P micronucleus with abnormal morphology and partial fusion (Figure 4L), while the main nucleus had a normal structure. In the other gonocyte, both the P genome nucleus and the R micronucleus exhibited normal morphology (Figure 4K). These images indicated selective elimination of the R chromatin and putative emergence of gonocytes with the P genome. This finding is novel, since we did not find corresponding metaphase plates containing predominantly the P genome. We also registered several gonocytes with mixed R/P micronuclei (Figure 4M).

The micronuclei varied in chromatin condensation, size and shape. We observed micronuclei with the same low condensation level as in the cell nucleus (Figure 4A,B,D–F,J–L), highly heterochromatic micronuclei (Figure 4C,D,G,H), with barely visible chromatin remnants (Figure 4C,I,J), and sometimes completely lacking the signal from either the *perezi* or *ridibundus* whole-genomic probe (Figure 4J). Generally, gonocytes contained 1 to 6 micronuclei, typically 1 or 2 near the main nucleus; more than 3 was rare. Notably, some gonocytes exhibited 5, 6, or even 7 micronuclei, which was highly unusual (Figure 4E,L).

*Frequency of micronuclei carrying various genome types*. Micronuclei were predominantly associated with the P subgenome, especially in crosses 6 (N = 171 micronuclei, P[%] = 77.2%) and 30 (N = 32 micronuclei, P[%] = 81.2%) (Table 2). R subgenome micronuclei were less frequent (17% in cross 6 and 18.8% in cross 30), while R/P mixed types were rare (5.8% in cross 6, none in cross 30) (Table 2, Figure 3C). Although the number of P micronuclei increased as the stage progressed (Figure 2A, lollipop plot), it did not align directly with the proportion of P chromosomes, peaking in stage category 40–42 before decreasing in 43–45, accounting for both crosses. In crosses 18 and 24, micronuclei composition was more balanced. Cross 18 (N = 17 micronuclei) showed 64.7% P, 29.4% R, and 5.9% R/P mixed micronuclei, while cross 24 (N = 62 micronuclei) displayed a notable increase in R micronuclei (37.1%) alongside 61.3% P and 1.6% mixed genome micronuclei (Table 2, Figure 3C). Both crosses exhibited rising P micronuclei numbers peaking at stage categories 37–42, followed by a drop at 43–45 (Figure 2C, lollipop plot). In stage 40 tadpoles from cross 24, the highest number of R-type micronuclei was recorded, accounting for 40% of all micronuclei at that stage (Table 2, Figure 3C). In cross 19 (N = 22 micronuclei), P micronuclei dominated (68.2%), with R micronuclei at 31.8% and no mixed types. The number of P micronuclei decreased from stage category 40–42 to 43–45 (Figure 2E, lollipop plot). Similarly, in cross 12 (N = 28 micronuclei), P micronuclei made up 60.7%, with R and R/P types each at 28.6% and 10.7%, respectively (Table 2, Figure 3C). Cross 12 showed a rise in P micronuclei from stages 31–33 to 37–39 (Figure 2G, lollipop plot), indicating a delayed peak compared to other crosses.

### 3.3. Genomes Passed Down from Adult P. grafi Males and Females

**Figure 5:**
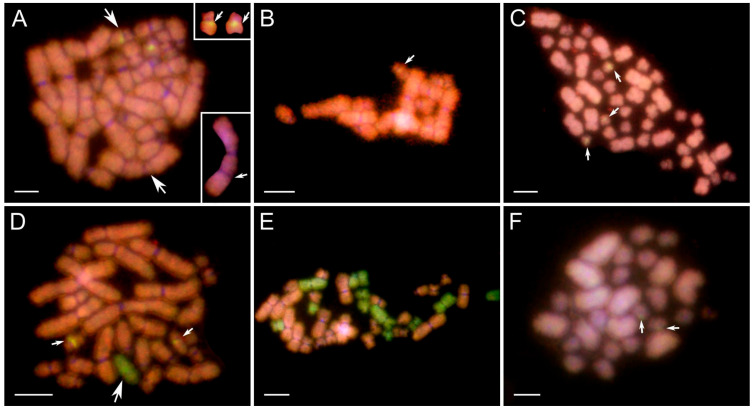
Chromosomal distribution and comparative genomic hybridization (CGH) analysis in SSC of adult hybrid males. (**A**) full diploid R metaphase plate (26 chromosomes) obtained from male no. 515. Big white arrows point to chromosomes cut into two insets: the upper inset includes two small R chromosomes with the pericentromeric signal of the *perezi* probe pointed by a small white arrow; the lower inset includes one big R chromosome with apparent double chromosomal breakage pointed by a small white arrow. (**B**) haploid R metaphase plate from male no. 509 with one small R chromosome displaying the pericentromeric signal of the *perezi* probe (shown by a small arrow). (**C**) metaphase plate with 44 R chromosomes (18 big, 26 small) from male no. 546 with three small R chromosomes displaying pericentromeric signals of the *perezi* probe (indicated by small arrows). (**D**) metaphase plate from male no. 515 with a diploid set of R chromosomes and an additional small P chromosome, two small R chromosomes have pericentromeric *perezi* probe signals (small white arrow). (**E**) metaphase plate from male no. 515 with 24 R chromosomes (10 big, 14 small) and 12 P chromosomes (3 big, 9 small). (**F**) metaphase plate from male no. 545 with 26 R chromosomes, among which two R chromosomes have a *perezi* probe signal. Scale bars 10 µm.

*Chromosomes in spermatogonial stem cells (SSCs)*. We examined 64 mitotic metaphase plates obtained from the gonads of 4 adult hybrid males (Supplementary Table S4). In all males, regular chromosomal complements consisting solely of R chromosomes predominated; however, many mitoses were haploid or aneuploid R (Figure 3B, Supplementary Table S5). Two males (545, 515) predominantly exhibited SSCs with proper genome elimination followed by endoreplication, resulting in diploid metaphases with 26 R chromosomes (Figure 5A,F), along with a small number of hypodiploid R metaphases. One of these males (515) had approximately one-third of metaphases with mixed R/P genome (Figure 5E), including diploid and hypodiploid, as well as triploid and hypotriploid RP configurations. In another male (509), diploid R complements accounted for more than half of all metaphases, with the remaining metaphases being haploid R. The male no. 546 showed a predominance of haploid and hypodiploid R metaphases, along with some diploid and hypertriploid R chromosome sets (3n = 39 with an additional 3 big and 2 small chromosomes; Figure 5C). Nearly all metaphase plates in four males contained small R chromosomes showing a green signal from the *perezi* whole-genomic probe in the pericentromeric region (Figure 5A–F), consistent with the signal observed in tadpole gonocytes. No micronuclei were observed in the interphase SSCs of the examined males. In all testes, prophase I meiocytes contained exclusively the R genome (*n* = 11). All studied males gave viable offspring (Table 1). Two males, 509 (crosses 16 and 17) and 515 (crosses 26–29), fathered the *P. ridibundus* tadpoles from the backcrosses. A high mortality was observed in tadpoles fathered by male 509. Males 545 and 546 were fathers of the crosses 30 and 6, respectively, described in Section 3.1 and Section 3.2 of this study.

*Lampbrush chromosomes in diplotene oocytes*. We analyzed 124 complete lampbrush chromosome sets from seven *P. grafi* females and one *P. perezi* female (Supplementary Table S12). To determine the genomic composition of oocytes, we examined chromosome morphology and performed FISH using telomeric probes. These probes revealed interstitial telomeric loci on chromosomes bearing the NOR region, which differed between the two parental species. Both interstitial sites in the P chromosomes were small, and *P. grafi* had the equivalent of R chromosomes with one large and one small site (Supplementary Figure S1C,D). All analyzed lampbrush chromosome sets from *P. grafi* hybrids were assigned to the R subgenome (Supplementary Figure S1A). Oocytes (*n* = 100) from six hybrid females had 13 R bivalents (Supplementary Figure S1). In one female (529), we found 23 oocytes with 13 R bivalents and one oocyte with 13 R univalents (Supplementary Figure S2).

*Taxonomic evaluation of the tadpoles from the backcrosses.* All six crosses (Table 1, nos. 16-17, 26-30) involving *P. grafi* hybrid parents exhibited high tadpole mortality. The highest mortality rates were recorded in crosses 16 and 27, with only nine tadpoles surviving to the preparation stage. RFLP-PCR analysis of all tadpoles (N = 89) revealed digestion patterns of *Rag1* and *Tyr1* sequences typical for *P. ridibundus* (Supplementary Table S2). These findings indirectly suggest that functional gametes from both male and female *P. grafi* transmit only the R genome, and that *P. grafi* × *P. grafi* matings can restore the second parental species, namely *P. ridibundus.*

## 4. Discussion

*Genome elimination in P. grafi gonocytes*. The F1 tadpoles from crosses between *P. perezi* and *P. grafi* carried a diploid RP genome, providing the first evidence that hybrids transmit the *ridibundus* (R) genome in their gametes and eliminate the *perezi* (P) genome. The P genome elimination was stage-specific: it started in gonocytes only at pro-metamorphosis and intensified during metamorphic climax but was not fully completed before the end of metamorphosis. Even at late tadpole developmental stages, some gonocytes retained uneliminated P chromosomes, suggesting that genome elimination and stabilization are extended processes and may remain unfinished in some individuals. Our former investigations on female *P. esculentus* showed that pregametogenesis is prolonged by about a year in comparison to the parental species [49,50]. However, the preliminary study on *P. grafi* gonad development showed that—contrary to *P. esculentus*—genome elimination does not cause such significant effects [51].

The rate and efficiency of the P chromosomes elimination varied across parental combinations, suggesting the influence of genetic and population-specific factors. We noticed that the most efficient P genome elimination occurred in crosses where hybrid mothers were donors of the R genome. In the primary cross between female *P. perezi* and male *P. ridibundus* (cross 12), gonocytes largely retained P chromosomes, with no clear evidence of systematic elimination. Such an effect has not been previously observed in hybrid offspring from primary *P. lessonae* × *P. ridibundus* crosses, where *P. esculentus* gonocytes preferentially eliminated one of the parental subgenomes, usually the *P. lessonae* genome [24,34,45,52]. We also observed differences in the elimination rates between large and small P chromosomes. In crosses with maternal transmission of the R genome (crosses 18, 19, 24), gonocytes more frequently eliminated large P chromosomes, whereas this pattern was absent in tadpoles with paternal R genome origin (crosses 6 and 30). To account for this, we hypothesize that maternally inherited factors, such as mitochondrial DNA or germplasm components deposited in the oocyte, may influence the elimination of the paternal P subgenome. These factors could be transmitted through zygotic and embryonic divisions into the germ cell lineage in yolk platelets, where primordial germ cells subsequently develop into gonocytes. It also appears likely that factors, including genomic compatibility or population-specific genomic interactions, modulate the efficiency of the elimination process [53].

*Micronuclei as carriers of the eliminated genome. Genomic composition of interphase gonocytes*. Micronucleus formation in *P. grafi* gonocytes represents a cytological mechanism of genome elimination in this hybrid, consistent with previous observations in *P. esculentus* [21,23,24,45,52]. A histological study of gonadal development in the parental species *P. perezi* revealed that male and female gonocytes lack micronuclei in their cytoplasm [51]. Most micronuclei in *P. grafi* were round-shaped, with chromatin condensation resembling that of the main nucleus, suggesting relatively intact chromatin packaging. Highly heterochromatic or very dispersed chromatin was found in a minority of micronuclei, possibly representing advanced stages of chromatin degradation followed by autophagy, corroborating findings in *P. esculentus* [21]. Micronuclei were frequently observed in tadpoles from all crosses involving a hybrid parent but were rare in tadpoles from *P. perezi* × *P. ridibundus* cross. Across all crosses, gonocytes in *P. grafi* tadpoles selectively eliminated the P subgenome, with micronuclei predominantly containing P chromosomes. However, the proportion of micronuclei with P chromatin varied among crosses. These results are consistent with the established model of hybridogenesis and gamete production in diploid adult individuals [2,4,54].

Nonetheless, in over half of the tadpoles, we detected micronuclei inconsistent with the expected pattern of hybridogenesis. Despite the substantial occurrence of R micronuclei in some crosses, tadpoles continued to efficiently eliminate the P subgenome, as reflected in the chromosomal composition of their gonocytes. This suggests the existence of distinct cellular clones within the gonads that follow divergent pathways of genome elimination. We propose that cells undergoing such non-selective elimination of the R genome are likely removed via apoptosis, since gonocytes with a predominance of P subgenome chromatin in their nuclei were only rarely observed. A similar phenomenon of both selective and non-selective genome elimination has been described in *P. esculentus*, where non-selective elimination led to cell death. At both early and late developmental stages, apoptosis was observed in gonocytes, spermatogonial stem cells (SSCs), and meiotic cysts in both ovaries and testes, frequently associated with partial or complete gonadal sterility [45,52,55]. In *P. grafi*, we also recorded a small fraction of micronuclei containing mixed R/P subgenomes. Based on studies in *P. esculentus*, which demonstrated that micronuclei typically enclose a single chromosome [24,45], we hypothesize that a similar pattern occurs in *P. grafi*. However, the co-occurrence of both genome types within a single micronucleus may indicate either the presence of two distinct chromosomes or randomly trapped chromosome fragments. It remains unclear whether the presence of both subgenomes within individual micronuclei is a phenomenon exclusive to *P. grafi* or if it also occurs in *P. esculentus*. Our previous studies of micronuclear genome elimination in *P. esculentus* employed FISH with a *P. ridibundus*-specific probe; therefore could not detect the simultaneous presence of *P. ridibundus* and *P. lessonae* chromosomes [24,45,52].

Typical *P. grafi* gonocyte nuclei were accompanied by one or two micronuclei, while cases with 3–7 micronuclei were relatively rare. This indicates that the intensity of chromosome elimination in individual cells of *P. grafi* is comparable to that observed in hybrid *P. esculentus*, which also eliminates 13 chromosomes [24,45,52]. In contrast, in hybridogenetic fishes of the genus *Hypseleotris*, gonocytes contained on average four micronuclei, with numbers ranging from 1 to 7, while these hybrids typically eliminate 22–24 chromosomes [11,56]. The observation that gonocytes lack a number of micronuclei consistent with the haploid chromosome set may indicate rapid micronuclear degradation through autophagy [21].

An intriguing finding in *P. grafi* was the spatial separation of the P and R subgenomes in numerous interphase gonocyte nuclei containing micronuclei. To date, such subgenomic compartmentalization has not been demonstrated in the germline of hybrid frogs [45], although it has been observed in gonocytes of hybrid *Hypseleotris* fish and in somatic cells during embryonic development of plant hybrids [56,57,58]. The spatial segregation of R and P chromatin in gonocytes may represent a preparatory mechanism for selective subgenome elimination, with the genome designated for removal positioned peripherally within the nucleus, adjacent to the nuclear envelope [57,58]; however, this hypothesis requires further validation.

*Genomic compositions of oocytes, spermatogenic stem cells (SSCs), and spermatocytes confirm accurate gametogenesis in adult P. grafi*. In hybrid tadpoles, most early meiotic prophase I oocytes contained mixed R/P genomes, with only a minority showing a pure R subgenome. Complete P subgenome elimination with subsequent R subgenome endoreplication occurred only in crosses 18 and 24. Some oocytes displayed dispersed chromatin threads of both subgenomes, likely reflecting asynapsis or synaptonemal complex defects. Research on mouse oocytes has demonstrated that synapsis failures trigger the DNA damage checkpoint, leading to the removal of affected oocytes during prophase I [59]. No direct data proves the same outcome for amphibians, and Dedukh et al. [31,52,60] showed instances of both parental genomes in diplotene oocytes in *P. esculentus*. Moreover, *P. esculentus* individuals frequently exhibit polyploidy, which introduces additional complexities in genome elimination, leading to oocytes with variable chromosomal compositions and the formation of triploid offspring [31,39,61]. Oocytes with mixed R/P genomes likely degenerated because in adult *P. grafi* females, lampbrush chromosomes in diplotene oocytes contained almost exclusively 13 R bivalents, supporting stable chromosomal compositions. Our results are consistent with the early study by Graf et al. (1977) [62], in which oocytes collected from adult *P. grafi* females exhibited enzyme protein isoforms characteristic only of the parental species *P. ridibundus*, indicating prior elimination of the *P. perezi* genome. SSCs of hybrid males in our study predominantly exhibited diploid R mitoses, although occasional aneuploid chromosomal sets or the simultaneous presence of R and P chromosomes indicated sporadic errors. Ultimately, it turns out that adult hybrids of both sexes produce gametes carrying the R genome. All tadpoles from *P. grafi* × *P. grafi* crosses (crosses 16, 17, 26–29) were genetically identified as *P. ridibundus* neoforms, confirming exclusive R genome transmission. SSCs with haploid 13 R chromosomes and oocytes with 13 univalents confirm separation between genome elimination and endoreplication processes. The uniformity observed in *P. grafi* contrasts with the heterogeneous spermatogenesis seen in diploid *P. esculentus* adult males, in which most SSCs carried both R and L parental genomes or displayed aneuploidy, leading to the subsequent cellular degeneration [45]. Highlighting the complexity of gametogenesis in these hybrids, the elimination of both parental subgenomes produced spermatozoa containing either haploid or diploid R sets, or alternatively, L chromosomes [31,33,36,37,45,63,64,65].

*Chromosome morphology in the germline cells*. Our CGH analysis of tadpole gonocytes and adult male SSCs revealed intact parental subgenomes, consistent with *P. grafi* somatic karyotypes [12]. However, some tadpole gonocytes and nearly all adult SSC metaphases displayed a small R chromosome with a *perezi* probe signal in the pericentromeric region of the long arm, mirroring the signal on the small P chromosome from pair 12. This germline-specific signal, absent from somatic cells [12], likely reflects chromatin remodeling rather than stable introgression, and its strong DAPI staining suggests AT-rich heterochromatin, possibly satellite DNA unmasked during germline epigenetic reprogramming [22,66,67,68,69].

*Potential threats to the stability of the P-G population*. The P-G (*P. perezi*-*P. grafi*) system closely parallels the western European L-E system (*P. lessonae*-*P. esculentus* diploid hybrids) [70], this study. A prevailing hypothesis proposes that, in *P. esculentus*, the clonally inherited R genome may gradually accumulate deleterious mutations, which become lethal when homozygous [71,72,73,74]. This significantly contributes to the stability of the L-E system [75]. A recent study by Burriel-Carranza et al. (2025) [76] reported the absence of *P. ridibundus* in Catalonian P–G populations, which they interpreted as evidence of the lethality of potential offspring from two *P. grafi* parents. Those populations are protected by a geographic barrier and have not yet experienced the invasion of *P. ridibundus* from southern France [75]. Although in this study, we demonstrated that experimental crosses between *P. grafi* individuals can reproduce *P. ridibundus*, this evidence deems it unlikely that such offspring could survive in natural environments. The P-G system may be disrupted by the rapid spread of the invasive *P. ridibundus*, resulting in mixed P-G-R populations and ultimately replacing native *P. perezi* and *P. grafi* populations at a high rate [20]. A computational model proposed for L-E populations by Bove et al. (2014) [75], where *P. esculentus* hybrids produce clonal R gametes, predicts a collapse of such systems when *P. ridibundus* individuals are introduced. Moreover, the geographical origin of *P. ridibundus* influences the type of F1 hybrid offspring: Southern European lineages produce sterile *P. esculentus*, while Central European ones yield fertile *P. esculentus* [77,78]. Pustovalova et al. (2024) [65] reported that *P. ridibundus* hybridizes with local *P. lessonae*, producing hybrids with reduced fertility and lacking genome elimination and endoreplication. Similarly, we found that hybrid tadpoles derived from primary crosses between native *P. perezi* and *P. ridibundus* were less efficient at eliminating the P subgenome. As studies underscore that the invasive *P. ridibundus* populations in France likely originate from multiple regions, including Eastern and Central Europe [18], this can be catastrophic for the P-G complexes’ existence. Although Hotz et al. (1994) [70] and our findings revealed a female-biased sex ratio, we cannot overlook the dangers of such invasion.

*Mechanisms of genome elimination in Pelophylax hybrids*. The determinants of selective genome elimination remain unclear, though maternal inheritance appears central. Most hybrid tadpoles in our study were female, supporting the maternal origin of the R subgenome. Gametogenesis in *Pelophylax grafi* is generally similar to that of the edible frog *P. esculentus* [21,24,31,33,37,39,45,79]. This parallel indicates that programmed genome elimination follows the same general pattern in different *Pelophylax* hybrid taxa, despite their independent hybrid origins. In this sense, the process can be considered evolutionarily conserved, as the non-*ridibundus* genome (in *P. grafi*, the P subgenome) is systematically susceptible to elimination, whereas the R genome is preferentially retained and transmitted to gametes. While it remains uncertain whether elimination is a single-step or gradual process, evidence suggests that gonocytes undergo extended G0/G1 arrest, with eliminated genomes expelled as micronuclei, later degraded by autophagy [22].

## 5. Conclusions

In *P. grafi* tadpoles, the P subgenome is selectively eliminated through micronuclei that emerge during gonocyte interphase, whereas the R subgenome is consistently retained and transmitted. The efficiency of genome elimination varies among parental combinations. It is enhanced in crosses with hybrid mothers transmitting the R genome, suggesting the influence of maternal inheritance and population-specific genomic interactions. Gonocytes that completely eliminate the P subgenome either undergo endoreplication of the R subgenome to restore diploidy and form functional gametes or fail to endoreplicate and remain haploid. Gonocytes with incomplete P subgenome elimination and with mixed R/P chromosomal sets degenerate after limited mitotic activity or during prophase of meiosis.

Micronuclei represent the primary mechanism of genome removal, with most containing P chromosomes, though occasional R or mixed R/P micronuclei indicate non-selective or incomplete elimination. Cells eliminating the R genome are likely to undergo apoptosis, preserving the overall accuracy of the hybridogenesis process. In adult *P. grafi*, germline genomes are more homogeneous, with diplotene oocytes and SSCs predominantly carrying R chromosomes, and gametes consistently transmitting only the R genome.

The data support a model in which genome elimination occurs during an extended G0/G1 arrest, with eliminated chromosomes expelled as micronuclei and degraded by autophagy. While molecular details remain unresolved, maternal inheritance and chromosomal architecture likely contribute to the regulation of genome elimination. Overall, *P. grafi* represents a system where hybridogenesis is stabilized by more efficient and accurate elimination of the P subgenome, contrasting with the higher variability observed in *P. esculentus*.

## Figures and Tables

**Table 1:** In vitro crosses were conducted between *P. grafi* and *P. perezi* frogs to produce progeny for cytogenetic analysis of genome elimination and genotype assessment in backcrosses. Cross numbers are highlighted in bold; the values in the brackets describe the number of tadpoles sacrificed from each cross for this study. Numbers in italic represent *P. grafi* × *P. grafi* backcrosses.

		Male							
Female		ID	546	510	513	509	548	515	545
		Genotype	RP	PP	RR	RP	PP	RP	RP
ID	Genotype	Population	Salagou	Montpellier	Montpellier	Montpellier	Salagou	Montpellier	Salagou
**521**	PP	Salagou			**12** (9)				
**532**	PP	Salagou	**6** (34)						
**524**	RP	Salagou							
**503**	RP	Montpellier		**18** (14)		***16*** (*5*)			
**520**	RP	Salagou		**19** (3)		***17*** (*20*)			
**525**	RP	Salagou						***27*** (*4*)	
**528**	RP	Salagou						***28*** (*20*)	
**531**	RP	Salagou					**24** (9)	***29*** (*20*)	
**501**	RP	Montpellier						***26*** (*20*)	
**537**	PP	Salagou							**30** (10)

**Table 2:** Frequencies of micronuclei in interphase gonocytes were analyzed in chromosomal preparations across different crosses and Gosner stage categories. The genome type of each micronucleus was determined based on genomic probe signals following the CGH procedure. The numerical values in the table represent the number of micronuclei in the given range. Micronuclei were categorized into three groups: P—*perezi* genome, R—*ridibundus* genome, R/P mixed—genomes of both species.

Cross no	Genome Type in Micronuclei	Gosner Stage Category					Percentage of Micronuclei [%]
		31–33	34–36	37–39	40–42	43–45	
		Micronuclei Count					
6	P	15	30	17	33	37	77.2
	R	1	10	6	8	4	17.0
	R/P mixed		3		3	4	5.8
12	P	6		11			60.7
	R	4		4			28.6
	R/P mixed	1		2			10.7
18	P		1	2	8		64.7
	R		1	1	3		29.4
	R/P mixed				1		5.9
19	P				15		68.2
	R				6	1	31.8
24	P			6	28	4	61.3
	R			5	17	1	37.1
	R/P mixed				1		1.6
30	P			7	18	1	81.2
	R			4	2		18.8

## Data Availability

The data underlying this article are available in the article and in its online supplementary material. Further inquiries can be directed at the corresponding author.

## Supplementary Materials

The following supporting information can be downloaded at https://www.mdpi.com/article/10.3390/biology14111526/s1, Supplementary File S1: 1. Chromosomal composition of gonocytes and oocytes in *Pelophylax grafi* tadpoles during gonadal development—detailed description. 2. Genomes passed down from adult *P. grafi* males and females—detailed description. Supplementary File S2: Figure S1. Lampbrush chromosomes from diplotene oocytes of *P. grafi* (A,C) and *P. perezi* (B,D). Figure S2. Lampbrush chromosomes from diplotene oocytes of *P. grafi* individual showing 13 bivalents (A) and 13 univalents (B). Supplementary Tables: Table S1. Adult individuals of water frogs from *Pelophylax perezi-grafi* system and *Pelophylax ridibundus* used in crossing experiments, cytogenetics and DNA extraction for whole-genomic probes. Table S2. Tadpoles of *Pelophylax perezi-grafi* system used in the study. Table S3. Tadpoles of *Pelophylax perezi-grafi* system used for CGH experiments. Table S4. Genomic composition of metaphase gonocytes in tadpoles and metaphase SSCs in adult males of *Pelophylax grafi*. Table S5. Ploidy levels and genomic composition of metaphase gonocytes in tadpoles and metaphase SSCs in adult males of *Pelophylax grafi*. Table S6. Ploidy levels and genome types of metaphase gonocytes in progeny of different crosses. Table S7. Genomic composition of meiotic prophase I oocytes in *P. grafi* tadpoles. Table S8. Descriptive statistics and analysis of differences in P and R chromosome drop rates between crosses using the Kruskal–Wallis ANOVA test. Table S9. Descriptive statistics and Mann–Whitney U test analysis of maternal and paternal R chromosome inheritance on P and R chromosome elimination rates. Table S10. Multiple regression analysis of maternal and paternal R chromosome inheritance on P and R chromosome elimination rates. Table S11. Analysis of genome eliminated within micronuclei in interphase gonocytes from *P. grafi* tadpoles. Table S12. Genomic composition of lampbrush chromosomes in oocytes of adult *P. grafi* and *P. perezi* females.
