# Supplementary material for: Programmed Genome Elimination Is Evolutionarily Conserved Across Pelophylax Hybrids—As Evidenced by P. grafi Hybridogenetic Reproduction
Source: Biology (Basel). 2025 Oct 30;14(11):1526. doi: 10.3390/biology14111526 (PMC12650731; doi:10.3390/biology14111526)
Supplement: Supplementary file 1 [file biology-14-01526-s001.zip › Supplementary File S1_Dudzik.pdf]

Programmed genome elimination is evolutionarily conserved across *Pelophylax* hybrids – as evidenced by *P. grafi* hybridogenetic reproduction

Anna Dudzik[1], Beata Rozenblut-Kościsty[1], Dmitrij Dedukh[2], Pierre-André Crochet[3], Lukas Choleva[4,5], Monika Przewłocka-Kosmala[6], Zuzanna Stryczak[1], Maria Ogielska[1], Magdalena Chmielewska[1]

Supplemental Results

1. Chromosomal composition of gonocytes and oocytes in *Pelophylax grafi* tadpoles during gonadal development – detailed description page 2

2. Genomes passed down from adult *P. grafi* males and females - detailed description page 5

1. Chromosomal composition of gonocytes and oocytes in *Pelophylax grafi* tadpoles during gonadal development – detailed description

Female *P. perezi* PP x male *P. grafi* RP

Chromosomal compositions of gonocytes from crosses 6 and 30. Tadpoles were analyzed at stages 31–45 (N = 44; 216 metaphase plates, Supplementary Tables S2-4). At stage category 31–33, 7 of 11 analyzed metaphase plates were diploid RP (Fig. 1A). Starting from stage 34, the number of R chromosomes remained constant (13 or 12), while the number of P chromosomes began to decrease, resulting in hypodiploid plates (Fig. 1B,D, also as presented in Fig. 1C,E,F for other crosses). In stages 39–44, haploid plates with 13 R chromosomes (as presented in Fig. 1G, cross 19) and diploid plates with 26 R chromosomes (as presented in Fig. 1I, cross 18 and 24) were observed. However, throughout all stages, in 15 mitoses the number of P chromosomes (6–13) was higher by up to 3 compared to the number of R chromosomes (4–12), suggesting the elimination of R subgenome or both P and R subgenomes.

Female *P. grafi* RP x male *P. perezi* PP

Chromosomal compositions of gonocytes from crosses 18, 19 and 24. Tadpoles were analyzed at Gosner stages 28–45 (N = 29; 167 metaphase plates, Supplementary Tables S2-4). The tadpoles from crosses 18 and 24 were characterized by more rapid P chromosome elimination than from cross 19. At stage 28, diploid RP chromosomal sets were found in 21 of 26 metaphase plates (as presented in Fig. 1A), and 3 mitoses were aneuploid with mixed genomes. At the stage categories 34–36 and 37–39, for 39 counted metaphase plates there were 6 metaphases with more than three P chromosomes. The remaining 33 metaphase plates had 3 or less P chromosomes, among which 6 were haploid R, one diploid R and one hypotriploid R. Among 103 metaphases from categories 40–42 and 43–45, 24 metaphases contained only R genome, including 13 diploid and 11 haploid. We found two peculiar hyperdiploid metaphase plates with 26 R chromosomes and 2 small P chromosomes (possibly from pair 6 or 7). Twenty-two metaphase plates were diploid RP, and 16 of them belonged to the tadpoles from cross no. 19. This cross exhibited a lower rate of P chromosome elimination, similar to crosses 6 and 30, and thus had a higher number of P chromosomes as compared to crosses 18 and 24. Therefore, the statistical analysis of these crosses was conducted separately. Throughout all stages, in 8 mitoses for cross 18 and 2 mitoses for cross 24, we recorded fewer than 13 R chromosomes. In those metaphase plates, P chromosomes ranged from 11 to 13, and R from 10 to 12 (with the

highest difference equal to three). Moreover, 95 metaphase plates were aneuploid R/P. In crosses 18 and 24, except for the majority of the female tadpoles, we also identified three male tadpoles and analyzed gonocytes from their testes. Their genomic compositions were similar to those of female tadpoles from the same crosses, which also showed *perezi* genome elimination (Fig. 1E). However, the data from these males were not included in the charts or statistical analysis.

Female *P. perezi* PP x male *P. ridibundus RR*

Chromosomal compositions of gonocytes from cross 12. The tadpoles obtained from this cross were analysed at stages 31-38 (N = 9, 77 metaphase plates, Supplementary Tables S2-4). Metaphase plates in stage categories 31-33 and 37-38 had a high number of P chromosomes, with 36 of them being diploid RP with no *perezi* genome elimination. Only 12 gonocytes had the number of P chromosomes lower than or equal to 10, while R chromosomes remained in a haploid state (13R). One metaphase plate was nearly haploid, with 1P and 13 R chromosomes (Fig. 1F).

Statistical summary of all the crosses pooled - detailed description

We performed the statistical analysis on 271 metaphase plates. Metaphase plates with less than or equal to 10 chromosomes, and those from three males, were excluded to provide a more refined and accurate representation of chromosomal drop rate dynamics, with differences in the significance of group comparisons. The Kruskal-Wallis ANOVA revealed a statistically significant difference in the drop rate percentage of P chromosomes (P% drop rate) among the crosses ($p < 0.001$), but not for the R chromosomes (R% drop rate) (Supplementary Table S8). We also divided and counted the big and small P and R chromosomes. The descriptive statistics for big (big P% drop rate) ($p < 0.001$) and small (small P% drop rate) ($p < 0.001$) P chromosomes showed differences within the groups. Analysis using the Kruskal-Wallis ANOVA identified specific intergroup differences for the P% drop rate among six analyzed crosses. Significant differences were observed between crosses: 6 and 12 ($p = 0.010$), 6 and 18 ($p < 0.001$), 6 and 24 ($p < 0.001$), 6 and 30 ($p < 0,005$), 12 and 18 ($p < 0.001$), 12 and 24 ($p < 0.001$), 12 and 30 ($p < 0.001$), 18 and 19 ($p < 0.001$), 18 and 24 ($p = 0.003$), 18 and 30 ($p < 0.001$), 19 and 24 ($p < 0.001$) and 19 and 30 ($p = 0.001$), 24 and 30 ($p = 0.001$) (Supplementary Table S8). Cross 19 showed no significant differences when compared with crosses 6 and 12. These results indicate a substantial variation in the drop rate of P chromosomes, with crosses

18 and 24 showing a consistently higher drop rate (Mean drop rate P% range 1.897-2.126) in relation to other crosses (Mean drop rate P% range 0.307-1.289), indicating they are more prone to P chromosome elimination. Significant differences in the drop rate of big and small P chromosomes were presented similarly to the overall P% drop rate (Supplementary Table S8). For the R% drop rate, the Kruskal-Wallis ANOVA did not find significant intergroup differences (Supplementary Table S8).

Next, we conducted descriptive statistical analysis for two subgroups based on the origin of the R subgenome (Supplementary Table S9). In one subgroup of crosses (nos. 18, 19 and 24) tadpoles inherited R chromosomes from the maternal side, and the other subgroup of crosses (nos. 6, 12 and 30) inherited R chromosomes from the paternal side. The subgroup with a maternal *P. grafi* (RP) origin exhibited a higher P% drop rate (mean of 1.44 ± 0.99) compared to the paternal *P. grafi* (RP) subgroup (mean of 0.99 ± 0.87). For the R% drop rate, the maternal *P. grafi* (RP) group had a mean of -0.01 ± 0.51, while the paternal *P. grafi* (RP) group had a mean of 0.09 ± 0.50. The Mann-Whitney U test demonstrated significant differences for the P% drop rate ($p < 0.001$), but not for the R% drop rate ($p = 0.598$), indicating that the parental origin of the R subgenome affects the P% drop rate. We also analyzed the drop rates of large and small P chromosomes within these subgroups which appeared different. In the subgroup with maternal origin of R chromosomes, the big P% drop rate was 1.48 ± 1.12, and the small P% drop rate was 1.42 ± 0.95. In the subgroup with paternal origin of R chromosomes both drop rates were lower; the big P% drop rate was 1.05 ± 1.00, and the small P% drop rate was 0.96 ± 0.87. The Mann-Whitney U test for big P% drop rate showed a significant difference ($p = 0.002$), suggesting that maternal origin of R chromosomes in crosses results in faster elimination of the *perezi* genome. Wilcoxon matched pairs test revealed statistically significant differences in the rate of decline between large and small chromosomes only in the group with maternal origin of R chromosomes: 1.48 ± 1.12 for big P% drop rate vs. 1.42 ± 0.95 for small P% drop rate ($p = 0.006$). In the group with paternal origin of R chromosomes the difference was not significant: 1.05 ± 1.00 for big P% drop rate vs. 0.96 ± 0.87 for small P% drop rate ($p = 0.056$). For the small P% drop rate, the Mann-Whitney U test was significant ($p < 0.001$), indicating variability across groups. This suggests that big P chromosomes are generally eliminated faster than small P chromosomes in both groups, but the effect is more pronounced when the R genome is maternally inherited. Additionally, regression analyses were performed to assess the association between the P% chromosomes drop rate and the origin of the R genome in tadpoles (Supplementary Table S10). A significant positive association was detected

($F(1,269) = 15.307$, $p < 0.001$), with a multiple R of 0.23 and adjusted R^2 of 0.05. This means that the drop rate of P chromosomes increases when R chromosomes are maternally inherited. For the R% drop rate, no significant association was observed ($F(1,269) = 2.775$, $p = 0.097$), with a multiple R of 0.10 and an adjusted R^2 of 0.0065. The regression coefficient was negative ($b = -0.1022$, $p = 0.097$), indicating a retention or increase of R chromosomes. However, given the *p*-value, this effect does not reach statistical significance.

2. Genomes passed down from adult *P. grafi* males and females - detailed description

Chromosomes in spermatogonial stem cells (SSCs). We examined 64 mitotic metaphase plates obtained from the gonads of 4 adult hybrid males (nos. 509, 515, 545 and 546) (Supplementary _Table S4). All males had SSCs with properly eliminated genome followed by endoreplication resulting in 26 diploid R mitoses (Fig. 5A,F). Seven diploid mitoses with R/P chromosomes were only found in one male (no. 515) (Fig. 5D,E). Eight haploid R metaphase plates (n=12, 13, or 14 chromosomes) were found in males nos. 509 and 546 (Fig. 5B). Male no. 545 exhibited the highest level of SSCs with diploid R mitoses. We documented only one metaphase plate with 44 R chromosomes (18 big and 26 small), which equals a hyper-triploid chromosome set (3n = 39 with an additional 3 big and 2 small chromosomes) (Fig. 5C). Nearly all but five metaphase plates had small R chromosomes with a green signal of the *perezi* whole-genomic probe in the pericentromeric region (Fig. 5A–F), which is similar to the signal found in tadpole gonocytes. All studied males gave viable offspring (Table 1). Two males, 509 (crosses 16 and 17) and 515 (crosses 26-29), fathered the *P. ridibundus* tadpoles from the backcrosses. Males 545 and 546 were fathers of the crosses 30 and 6, respectively, described in part 1 of this study. A high mortality was observed in tadpoles fathered by male 509. We did not observe any micronuclei in the interphase SSCs of the described males. In all male testes we found prophase I meiocytes containing R genome: one in male 509, two in male 515, three in male 545 and five in male 546. Most SSCs exhibited regular 2n R chromosomal sets, though some cells exhibited irregular ploidy (Fig. 3B, Supplementary Table S5). Male 509 had 54.55% diploid R and 45.45% haploid R metaphase plates. In male 515, 61.91% of the metaphase plates were diploid R, 9.52% diploid RP, 4.76% triploid RP, 4.76% hypodiploid R, 9.52% hypodiploid RP and 9.52% hypotriploid RP. Male 545 showed 96.15% diploid R and 3.85% hypodiploid R metaphase plates. Finally, in male 546, 44.44% metaphase plates were haploid R, 11.11% diploid R, 33.33% hypodiploid R, and 11.11% hypertriploid R.
