# Supplementary material for: Programmed Genome Elimination Is Evolutionarily Conserved Across Pelophylax Hybrids—As Evidenced by P. grafi Hybridogenetic Reproduction
Source: Biology (Basel). 2025 Oct 30;14(11):1526. doi: 10.3390/biology14111526 (PMC12650731; doi:10.3390/biology14111526)
Supplement: Supplementary file 1 [file biology-14-01526-s001.zip › Supplementary File S2_Dudzik.pdf]

**Hybridogenesis in the Natural Hybrid Frog *Pelophylax grafi*. How Precise is the Genome Elimination from the Germline Cells?**

Anna Dudzik[1], Beata Rozenblut-Kościsty[1], Dmitrij Dedukh[2], Pierre-André Crochet[3], Lukas Choleva[4,5], Monika Przewłocka-Kosmala[6], Zuzanna Stryczak[1], Maria Ogielska[1], Magdalena Chmielewska[1]

**Supplementary Figure S1** page 2

**Supplementary Figure S2** page 4

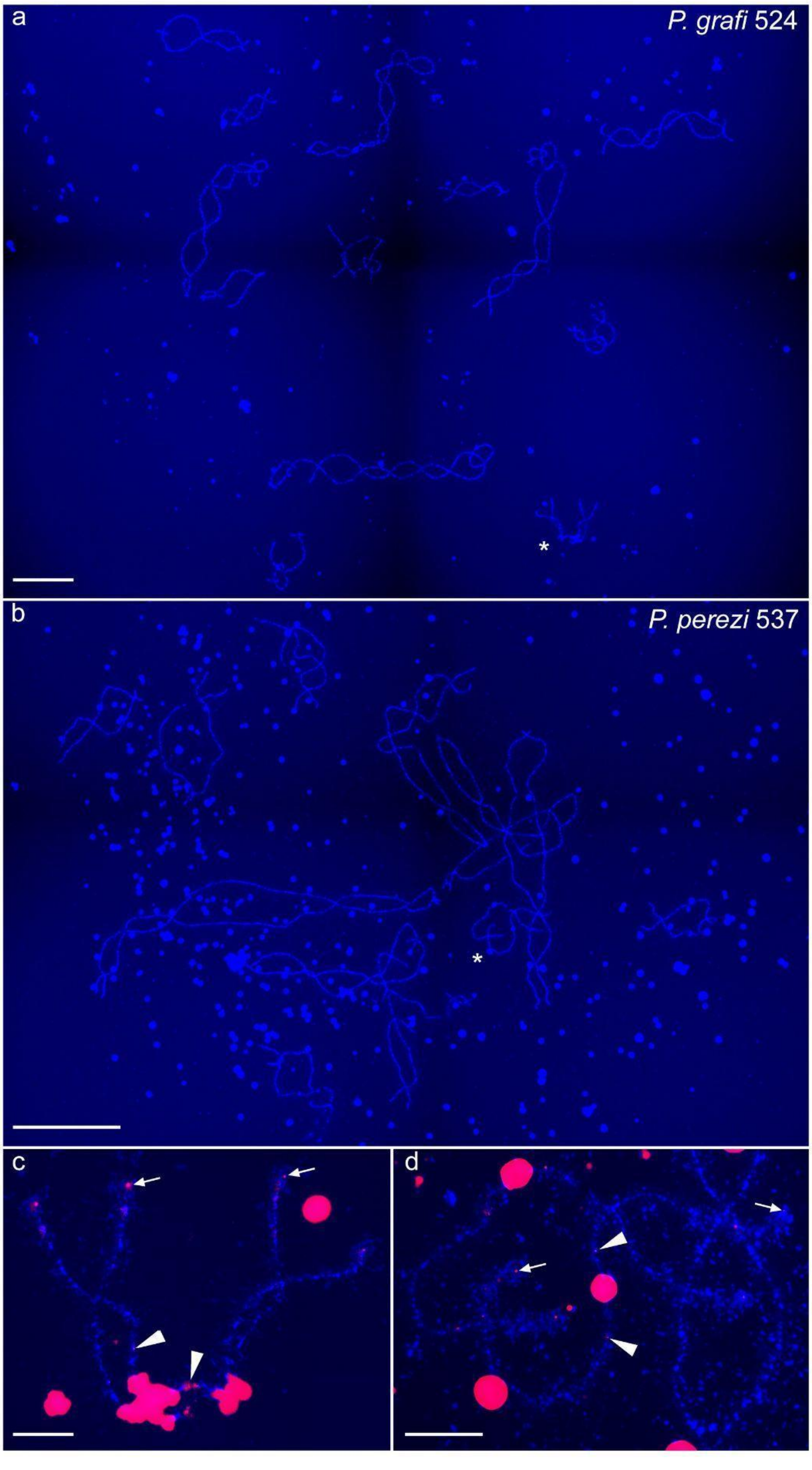
a
P. grafi 524
b
P. perezi 537
c
d

**Supplementary Figure S1. Lampbrush chromosomes from diplotene oocytes of *P. grafi* (A, C) and *P. perezi* (B, D).** Lampbrush chromosomes of *P. ridibundus* in *P. grafi* oocytes (A) and *P. perezi* lampbrush chromosomes (B) are represented by 13 bivalents. Chromosomal spreads from the individual oocytes were large and required four images, which were subsequently merged into a single image. Enlarged lampbrush chromosomes H (indicated by asterisks in A, B) are presented in C, D after FISH with (TTAGGG)n probe. In *P. ridibundus* lampbrush chromosome H, small and large sites of interstitial (TTAGGG)n repeat (indicated by arrowheads, red) were identified (C). In *P. perezi* lampbrush chromosome H, both sites of interstitial (TTAGGG)n repeat (indicated by arrowheads, red) were small. Arrows indicate telomeric regions (red) of individual bivalents. Scale bars: (A) 50μm, (B) 10μm.

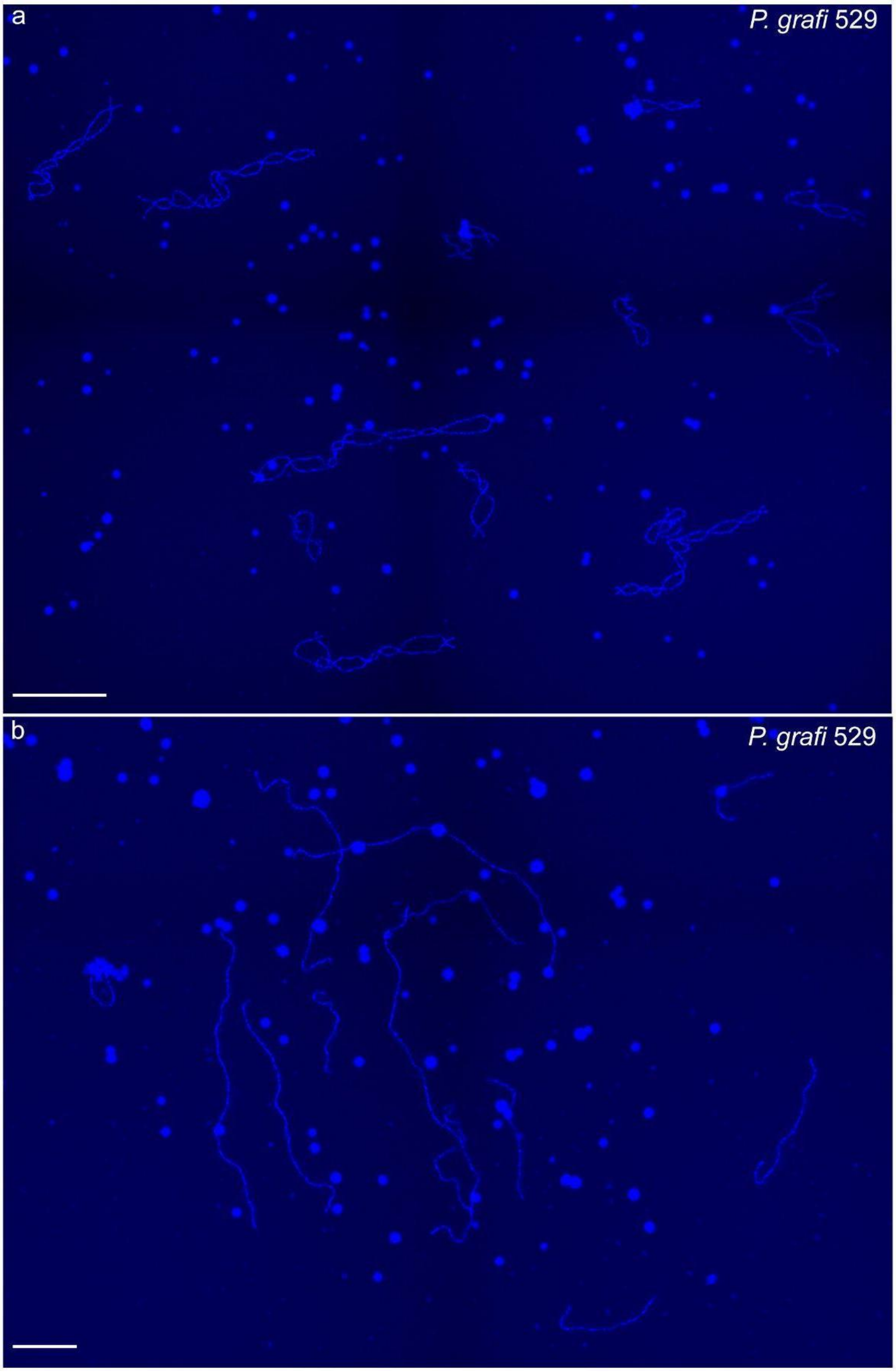


**Supplementary Figure S2. Lampbrush chromosomes from diplotene oocytes of P. grafi individual showing 13 bivalents (A) and 13 univalents (B).** Chromosomal spreads from the individual oocyte were large and required four images, which were subsequently merged. Scale bars: 50µm.
